# Mr. Ring, on Mr. Pears's Cases

**Published:** 1802-09-01

**Authors:** John Ring


### *To the Editors of the Medical and Phyſical Journal.*

GENTLEMEN,

I Beg leave to offer a few Remarks on the Caſes publiſhed in the laſt Number of your Journal, by Mr. Pears, to whom I return thanks for the manner in which he ſpeaks of my attempts to promote Vaccine Inoculation; but I can ſcarcely coincide in opinion with him, that it was neceſſary to bring forward theſe caſes in the Medical Journal.

Such rumours as thoſe which Mr. Pears alludes to, when left to themſelves, gradually die away. Any notice of them in print, only ſerves to revive them.

The account publiſhed by Mr. Pears, would make any one ſuppoſe the puſtulous eruption in Mary Salt, much more formidable than it really was. Of this I need bring no better proof than that adduced by Mr. Pears himſelf, who ſays, Mr. Heaumes, the gentleman who attended her, informed him there was *no fluid*; and the mother of the child told him the puſtules contained a *watery fluid*. Mr. Heaumes alſo ſaid, the accompanying raſh reſembled the meaſles.

Mr. Pears is miſtaken in his opinion concerning the other child; it was not inoculated by me. This, however, is of but little moment, ſince it is well underſtood, that neither of them has had the ſmall-pox.

Mr.

Mr. Heaumes having ſeen Mary Salt, thought the eruption of the variolous kind. He acknowledged, however, in his converſation with me, the ſame evening, that in ſome places it appeared more like the meaſles.

The whole appeared to me of the herpetic kind, which was the opinion I expreſſed; but being informed that the ſmall-pox had been in the houſe; that three medical men had pronounced this to be that diſorder; that two other children who had been vaccinated in that neighbourhood, had ſickened for the ſmall-pox, and that a variolous eruption began to appear on one of them, I could not but entertain a doubt whether the patient had in reality had the perfect cow-pox. This doubt was ſtrengthened by the following circumſtance: Of three patients inoculated with matter taken from this child, infection took place only in one; and in that inſtance, the puſtule did not begin to appear till the fifteenth day.

Another of the children, who was ſuppoſed to be ſickening for the ſmall-pox, had been eating too much fruit, and had twenty-ſix ſtools within twenty-four hours. I gave my opinion, that nothing was to be feared reſpecting the ſmall-pox in this caſe. The diarrhœa was removed by the next morning; and when I called on this patient, I was informed by her mother, that Mary Salt had riſen at her uſual time, and complained of hunger; that her raſh was much abated; and that all alarm concerning her having the ſmall-pox was nearly at an end. This account I had the pleaſure to find, on calling at the houſe, was true. From that time, which was the *second*, and not the *fourth* day of the eruption, it is well known to the mother of the child, and to ſeveral of the neighbours, I gave my opinion that the caſe was not the ſmall-pox; I alſo communicated the ſame opinion to Dr. Jenner.

It is well known, that the ſmall-pox is a proteiform diſeaſe, aſſuming almoſt every colour and ſhape. Hence, it follows that there is no complaint with which the human body is afflicted, that has given riſe to more miſtakes. Errors of this kind are continually occurring; and if you wiſh for caſes of this ſort, it would be no difficult matter to collect a ſufficient number to fill a Journal once a month.

Neverthele ſs, I am far from concluding, that any diſcuſſion of this kind will be of final diſadvantage to the new practice, ſince it will probably occaſion profeſſional men to be a little more cautious in giving a deciſion concerning eruptive diſorders, when their opinions are likely to be brought before the tribunal of the public.

I now ſhall add a few remarks on another article in the ſame number of your valuable Journal, by Mr. Badger.

To

To me it appears, that the eruptive difeafe alluded to by Mr. Badger, is the herpes puftulofus of Bell; an account of which may be feen in his Treatife on Ulcers. Eruptions of this kind, he obferves, appear moft frequently on the face, behind the ears, and on other parts of the head; and occur moft commonly in children.

I have often feen a puftulous eruption affecting the fcalp, but not always confined to the fcalp, at different periods, from the earlieft infancy to the age mentioned by your correfpondent; but never had the leaft reafon to fufpect it to be infectious, unlefs by actual contact. When conveyed by the teeth of a comb, it is generally underftood, that the matter of this difeafe may communicate infection from one perfon to another. I have alfo frequently feen the fame complaint in thofe more advanced in years. One cafe, extremely violent in its nature, lately fell under my care, in a young woman, feventeen years of age, the daughter of Mr. Webb, at the Griffin, in Half Moon Street.

As to the treatment of the complaint, it is certainly improper to apply ointments while the puftules continue; and I am warranted by experience to fay, that mercurials have no advantage over other catharics in this difeafe. The practice of Mr. Badger, as well as my own, authorizes me to conclude, that, as far as regards internal remedies, there is no fpecific in this complaint. If an inflammatory diathefis prevail, the antiphlogiftic plan fhould be followed; if debility fhould appear, the tonic plan is indicated. If the hair be not removed, tinea capitis will enfue.

I have lately conveyed vaccine matter on a thread well impregnated, and enclofed in a tobacco-pipe, ftopped with wax at each end, to many diftant parts. Dr. Domeier, Phyfician to his Royal Highnefs the Duke of Suffex, at different times, received two parcels from me while at Lifbon, both of which proved fuccefsful. Some of the produce of this matter he fent to Madrid, where it alfo fucceeded; and to the Brazils. I have likewife fent virus on a tooth-pick to feveral places, and received favourable reports. A puncture is firft to be made with a lancet; then the tooth-pick to be inferted, and retained fome time in the puncture.

I am, &c.

JOHN RING.

